# Modelling the cost‐effectiveness of a chloramine gel in treating infected, non‐healing diabetic foot ulcers

**DOI:** 10.1002/hsr2.70076

**Published:** 2024-10-28

**Authors:** Julian F. Guest, Björn Eliasson

**Affiliations:** ^1^ Catalyst Consultants Poole UK; ^2^ Department of Medicine Sahlgrenska University Hospital Gothenburg Sweden

**Keywords:** chloramine, chlorasolv, cost‐effectiveness, debridement, diabetic foot ulcer, UK

## Abstract

**Background and aims:**

Organic chloramines have been developed as a topical wound bed preparation gel. This study aimed to estimate whether the addition of chloramine gel (Chlorasolv, RLS Global AB, Sweden) to standard care compared with standard care alone would afford a cost‑effective technology to the UK's health services for treating infected, non‐healing diabetic foot ulcers (DFUs).

**Methods:**

A Markov model was developed to simulate the management of infected, non‐healing DFUs. The model utilised data from a randomised controlled trial and was used to estimate the cost‐effectiveness of chloramine plus standard care compared with standard care alone over a period of 24 weeks, expressed as the incremental cost per quality‐adjusted life year (QALY) gained at 2021/22 prices.

**Results:**

Using adjunctive chloramine to treat infected, non‐healing DFUs was found to shorten the time to healing by 36% (from a mean of 17.1 weeks per ulcer to a mean of 11.0 weeks per ulcer). This translated into a 6% improvement in the probability of being healed by 24 weeks and a corresponding 3% improvement in health‐related quality of life (HRQoL). Also, use of adjunctive chloramine was found to reduce the total cost of ulcer management by 13%. Sensitivity analysis found that adjunctive chloramine remained a cost‐effective treatment, even when the value of the model inputs was varied by ±20%.

**Conclusion:**

Within the limitations of the study, treatment with adjunctive chloramine instead of standard care alone could potentially afford the UK's health services a cost‐effective debridement strategy for infected, non‐healing DFUs, due to its ability to accelerate the time to healing.

## 1 INTRODUCTION

The lifetime risk of people with diabetes developing a diabetic foot ulcer (DFU) is estimated at 19% to 34%, and this estimate is expected to rise with increasing longevity^1^ and increasing diabetic neuropathy or peripheral arterial disease.^2^ DFUs are a major source of morbidity often leading to deteriorating functional status, infection, lower extremity amputation and death.^3^ In a population‐based study, the incidence of a first‐time DFU was estimated at 0.8% and the overall incidence of foot ulceration was 1.1%.^4^

Over 50% of all DFUs become infected^5^, ^6^ and an estimated 20% of moderate or severe diabetic foot infections lead to some level of amputation.^7^ Hence, averting infection requires prompt and regular assessment and management.^8^ Ulcers that become infected are likely to contain treatment‐resistant biofilm, contributing to them becoming non‐healing.^9^, ^10^ Notwithstanding, peripheral arterial disease and peripheral neuropathy are independent risk factors for infection and non‐healing.^7^

First‐line therapies for DFUs include early referral for multidisciplinary care, debridement of necrotic tissue, pressure offloading, treating ischaemia and infection.^11^, ^12^ Guidelines for treating DFUs also recommend the application of an appropriate dressing.^13^ However, there are minimal differences in the published efficacy between many of the dressings in current use, due in part to the lack of robust data,^2^, ^14^ and new treatment options have generally failed to show clinical or cost‐effective superiority compared with modern standards of care,^15^ with a few exceptions.^16^, ^17^ Many ulcers present with superficial infections, which can be treated effectively with antibiotics. However, some require surgical intervention to remove deep soft tissue infection.^11^ The use of negative pressure wound therapy and topical oxygen therapy has recently been recommended in wounds that fail to respond to standard debridement.^18^, ^19^

Organic chloramines have antiseptic and antibacterial properties and have been developed as a topical wound bed preparation gel (Chlorasolv, RLS Global AB, Sweden) for cleansing and debriding/desloughing non‐healing venous leg ulcers and DFUs.^20^, ^21^ In one randomised controlled trial (RCT) in Sweden, 37 adult patients (mean age of 70 years) with type 1 or type 2 diabetes, with an infected, non‐healing DFU were randomised to receive either the chloramine‐based gel plus standard care or standard care alone.^21^ Patients in both groups were seen at least once a week for 12 weeks, but were followed (and treated, if needed) for 24 weeks.^21^ Patients had their diabetes for more than 20 years and their foot ulcer for ~1.5 years.^21^ After 5 weeks of treatment, there was a significant difference in relative reduction in ulcer area (*p* = 0.016) between the two groups in the intention‐to‐treat (ITT) cohort.^21^ After 9 weeks, 37% of patients (*n* = 7) had healed in the chloramine‐treated group, but only 6% (*n* = 1) in the control group (*p* = 0.039) in the ITT cohort.^21^ However, there was no statistically significant difference in ulcer healing at 24 weeks, and no marked differences in signs of infection, pain, quality of life or incidence of adverse events.^21^ The authors concluded that debridement with the chloramine gel led to a more rapid transformation from necrotic/devitalised tissue to purulence and red granulation than that seen with standard care.^21^

This health economic study aimed to estimate whether the chloramine gel potentially affords a cost‑effective technology to the UK's publicly‐funded health services for treating infected, non‐healing DFUs, using the ITT cohort in the Swedish RCT as the clinical basis for the analysis.

## 2 METHODS

### 2.1 Study design

This was a health economic modelling study based on a retrospective cohort analysis of the anonymised case records of the 37 patients who had an infected, non‐healing DFU in the aforementioned RCT.^21^ Ethical approval for this economic study was included in the approval to perform the RCT in Sweden by The Regional Ethics Review Board (Dnr 317‐13).

### 2.2 Economic modelling

**Figure 1:**
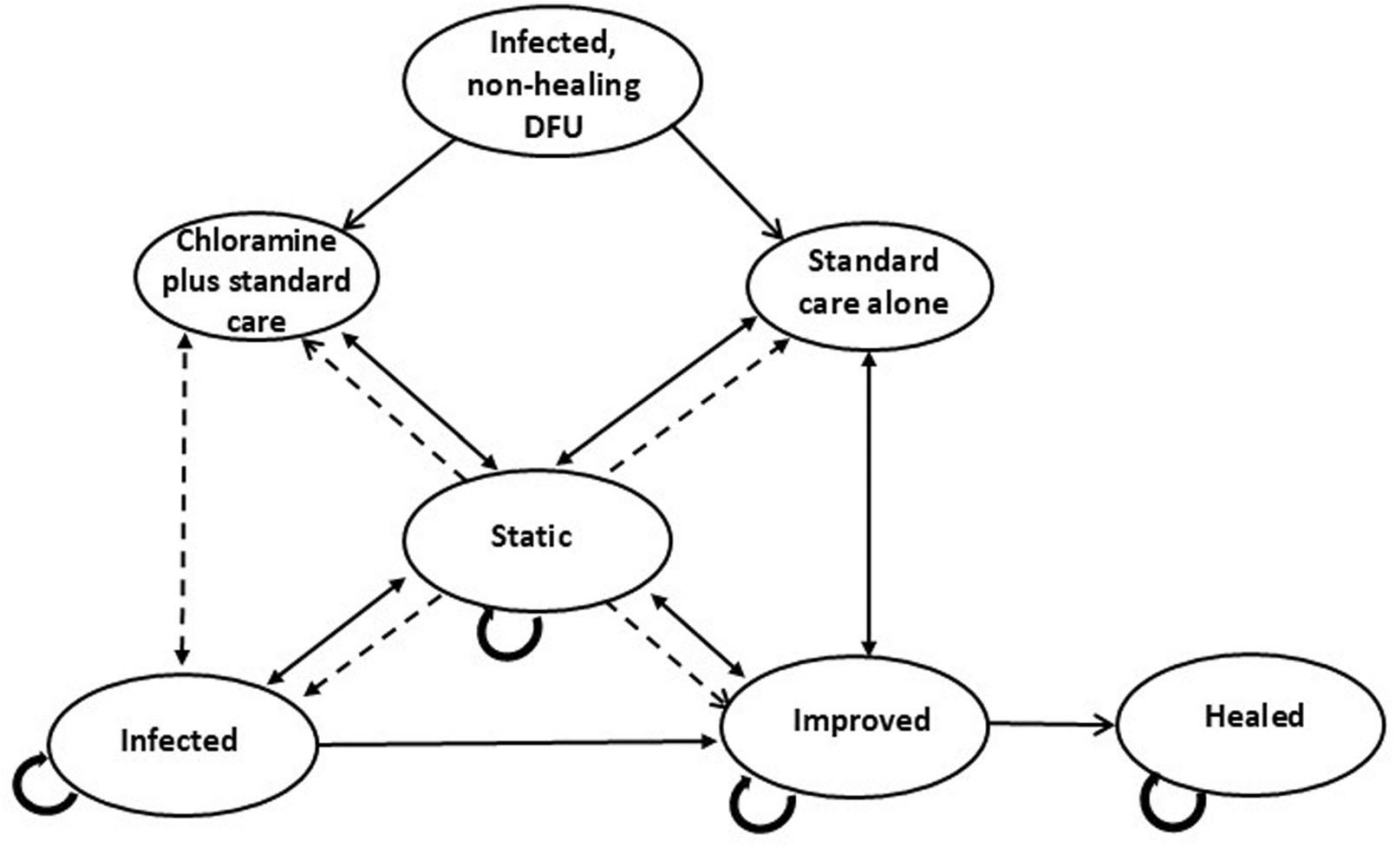
Diagrammatic representation of the Markov model.

A Markov model (Figure 1) was constructed to assess the incremental cost‐effectiveness of adjunctive chloramine compared with standard care alone in the treatment of infected, non‐healing DFUs. The model simulated the management of a cohort of patients of a mean age of 70 years with type 1 or type 2 diabetes who had an infected, non‐healing DFU for a mean of ~1.5 years. The model was built in MS Excel and comprised four health states: static (if the ulcer size had not decreased), improved (if the ulcer size decreased), healed and infected.

Patients enter the model and receive one of two treatments: adjunctive chloramine or standard care alone. Patients then transition with varying probabilities to one of three states: static, infected or improved. They can then remain in that state or move with varying probabilities to one of the other states, transitioning in weekly cycles for 24 weeks.

**Figure 2:**
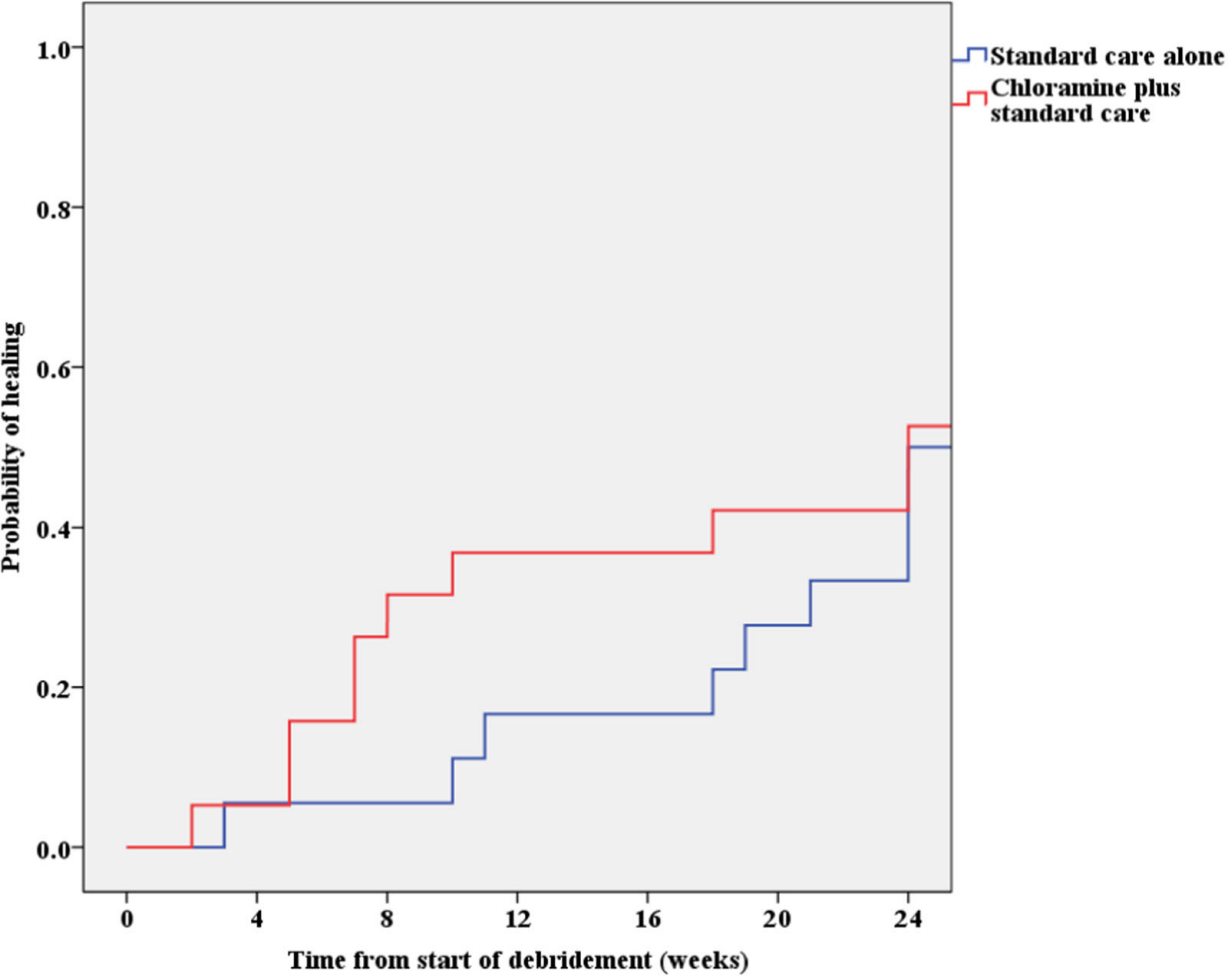
Kaplan‐Meier probability of healing analysis.

During the RCT, patients were treated at least once a week for 12 weeks, although they were followed and treated, if needed, for 24 weeks.^21^ Weighted moving averages were used to interpolate any missing ulcer sizes between 12 weeks and 24 weeks of follow‐up for each patient. Kaplan‐Meier analysis was used to support this (Figure 2). The resulting rates of ulcer healing, improvement, remaining static and infection among the ITT cohort in the RCT were used to estimate the probabilities of patients transitioning between health states over the time horizon of the model (Table 1). A cycle length of 1 week was selected because ulcer management in the RCT took place in the clinics at weekly intervals. The model terminated after 24 weeks since that was the period of follow‐up in the RCT.^21^

**Table 1:** Transition probabilities at 4‐weekly intervals.

Week	Treatment	Static ulcer	Improved ulcer	Healed ulcer	Infected ulcer
0	Chloramine plus standard care	0.21	0.00	0.00	0.79
4	Chloramine plus standard care	0.16	0.32	0.05	0.47
8	Chloramine plus standard care	0.26	0.21	0.32	0.21
12	Chloramine plus standard care	0.05	0.32	0.37	0.26
16	Chloramine plus standard care	0.11	0.47	0.37	0.05
20	Chloramine plus standard care	0.11	0.42	0.42	0.05
24	Chloramine plus standard care	0.11	0.32	0.53	0.05
0	Standard care alone	0.11	0.00	0.00	0.89
4	Standard care alone	0.28	0.17	0.06	0.50
8	Standard care alone	0.28	0.33	0.06	0.33
12	Standard care alone	0.28	0.17	0.17	0.39
16	Standard care alone	0.11	0.67	0.17	0.06
20	Standard care alone	0.11	0.56	0.28	0.06
24	Standard care alone	0.09	0.33	0.50	0.07

### 2.3 Healthcare resource use

Standard care comprised the combination of dressings, nonsurgical debridements and offloading devices administered to patients in the ITT cohort in the RCT. The mix of dressings comprised a combination of foams, hydrocolloids or alginates. In a few cases an antiseptic agent, silver or PHMB dressings was also used.

The number of clinician visits, dressings, debridements and offloading devices that patients in the ITT cohort received in the RCT was quantified for each heath state. These valuations were used to populate the model's health states with relevant resource use estimates.

The model assumed that the frequency of clinician visits and the standard care that patients received in the RCT were comparable to the care pathway implemented in clinical practice in the UK. The model also assumed that both groups would be managed by the following ratio of healthcare professionals:

- District nurses – 20%
- Practice nurses – 20%
- Tissue viability nurses – 20%
- Podiatrists – 20%
- Hospital‐based multidisciplinary team – 20%

In the model, chloramine was applied according to the estimated applications administered to the ITT cohort in the RCT (Table 2). Additionally, antibiotic use in the model reflected the estimated prescriptions dispensed to the ITT cohort in the RCT.

**Table 2:** Chloramine dosing schedule estimated from the ITT cohort in the RCT.^21^

	Mean number of weekly chloramine applications		
Week	Improved ulcer	Static ulcer	Infected ulcer
0		1.0	1.0
1	0.6	1.0	1.0
2	0.5	1.0	1.0
3	0.4	1.0	1.0
4	0.3	1.0	1.0
5	0.7	1.0	1.0
6	0.7	1.0	0.6
7	0.7	1.0	0.6
8	0.7	1.0	0.6
9	0.7	1.0	0.6
10	0.7	1.0	0.6
11	0.7	0.5	0.6
12	0.7	0.5	0.6

### 2.4 Utilities

Patients’ health‐related quality of life (HRQoL) in terms of utilities were not recorded during the RCT. Therefore, published utility scores for DFUs (0.465 for an unhealed ulcer, 0.465 for an infected ulcer, 0.60 for a healed ulcer) were assigned to each health state in the model.^22^ These scores were used to estimate patients’ expected HRQoL in terms of the number of quality‐adjusted life years (QALYs) over 24 weeks.

### 2.5 Unit costs

Unit costs in GB pounds sterling at 2021/22 prices^23^, ^24^, ^25^ were applied to the resource utilisation estimates in the model (Table 3), enabling the cost of ulcer management to be estimated over 24 weeks. The time horizon of the model was less than 1 year, therefore a discount rate was not applied.

**Table 3:** Unit costs at 2021/22 prices.^23^, ^24^

Resource	Unit Cost
Practice nurse visit	£26.00
District nurse visit	£55.00
Tissue viability nurse visit	£95.32
Podiatry visit	£76.07
Hospital outpatient visit with the multidisciplinary team	£224.05
Chloramine (per administration)	£49.00

### 2.6 Model outputs

The model's outputs were:

- Patients’ HRQoL in terms of the number of QALYs at 24 weeks.
- The probability of being healed by 24 weeks.
- The total cost of ulcer management over 24 weeks.
- The relative cost‐effectiveness of chloramine plus standard care.

#### 2.6.1 Cost‐effectiveness analysis

The potential cost‐effectiveness of adjunctive chloramine compared with standard care alone was calculated as ‘the difference between the total cost of ulcer management of the two groups’ ÷ ‘the difference in the number of QALYs between the two groups’. The resultant incremental cost‐effectiveness ratio (ICER) was expressed as the incremental cost per QALY gained. A treatment was considered to be a dominant (cost‐effective) intervention if it generated more QALYs for less cost.

#### 2.6.2 Sensitivity analysis

Probabilistic sensitivity analysis (PSA) was performed by assigning a distribution to the deterministic values (beta distributions for probabilities and utilities and gamma distributions for resource use and costs) and assuming a standard error of 20% around the mean values, since that was considered the limit of the plausible range of values. 10,000 iterations of the model were undertaken by random selection of a value from all the model inputs simultaneously. The output from this analysis was a distribution of costs and QALYs at 24 weeks, from which the probability of adjunctive chloramine being cost‐effective at different cost per QALY thresholds was estimated.

Deterministic sensitivity analysis in the form of a tornado diagram was undertaken to assess the effect of separately changing the values of individual model inputs. These inputs were varied by up to ±20% around the model's base case values since that was considered the limit of the plausible range of values.

## 3 RESULTS

### 3.1 Clinical outcomes and healthcare costs

Use of adjunctive chloramine shortened the time to healing by 36%, from a mean of 17.1 ± 7.5 weeks per ulcer to a mean of 11.0 ± 8.0 weeks per ulcer (Table 4). This resulted in a 6% improvement in the probability of being healed by 24 weeks. There was also a cumulative gain in QALYs associated with a 3% improvement in HRQoL, due to the accelerated change in healing (Table 4). Hence, treating an infected, non‐healing DFU with adjunctive chloramine affords a clinically more effective strategy than standard care alone.

**Table 4:** Clinical outcomes from the model.

	Chloramine plus standard care	Standard care alone
Mean time to healing (weeks)	11.0 ± 8.0	17.1 ± 7.5
Probability of having a healed ulcer at 24 weeks	0.53	0.50
Probability of having an improved ulcer at 24 weeks	0.32	0.33
Probability of having an unchanged ulcer at 24 weeks	0.11	0.09
Probability of having an infected ulcer at 24 weeks	0.05	0.07
Mean number of cumulative QALYs at 24 weeks	0.295	0.287

The total cost of ulcer management was estimated to be £5032 per DFU over 24 weeks in the adjunctive chloramine group and £5775 per DFU in the standard care group (Table 5). Hence, use of adjunctive chloramine was found to reduce the total cost of ulcer management by 13%. Hospital outpatient visits accounted for up to 38% of the total cost of ulcer management. Dressings accounted for up to 16% of the total cost and chloramine for a further 8%.

**Table 5:** Costs of healthcare resource use per DFU over 24 weeks from the model.

	Chloramine plus standard care		Standard care alone	
Hospital outpatient visits	£1827.78	(36%)	£2166.39	(38%)
Tissue viability nurse visits	£777.61	(15%)	£921.67	(16%)
Dressings	£573.23	(11%)	£904.94	(16%)
Podiatry visits	£620.57	(12%)	£735.54	(13%)
District nurse visits	£448.68	(9%)	£531.81	(9%)
Chloramine	£407.99	(8%)	£0.00	(0%)
Practice nurse visits	£212.11	(4%)	£251.40	(4%)
Antibiotics	£164.21	(3%)	£263.02	(5%)
TOTAL	£5032.18	(100%)	£5774.77	(100%)

*Note*: Percentage of total cost in parentheses.

### 3.2 Cost‐effectiveness analysis

Use of adjunctive chloramine was found to reduce the total cost of ulcer management by £743 over 24 weeks and increase a patient's HRQoL by 0.008 QALYs. Hence, including chloramine into a standard care protocol could potentially afford the UK's health services a cost‐effective intervention for treating infected, non‐healing DFUs since it improves outcomes for less cost.

### 3.3 Sensitivity analyses

#### 3.3.1 Probabilistic sensitivity analyses

**Figure 3:**
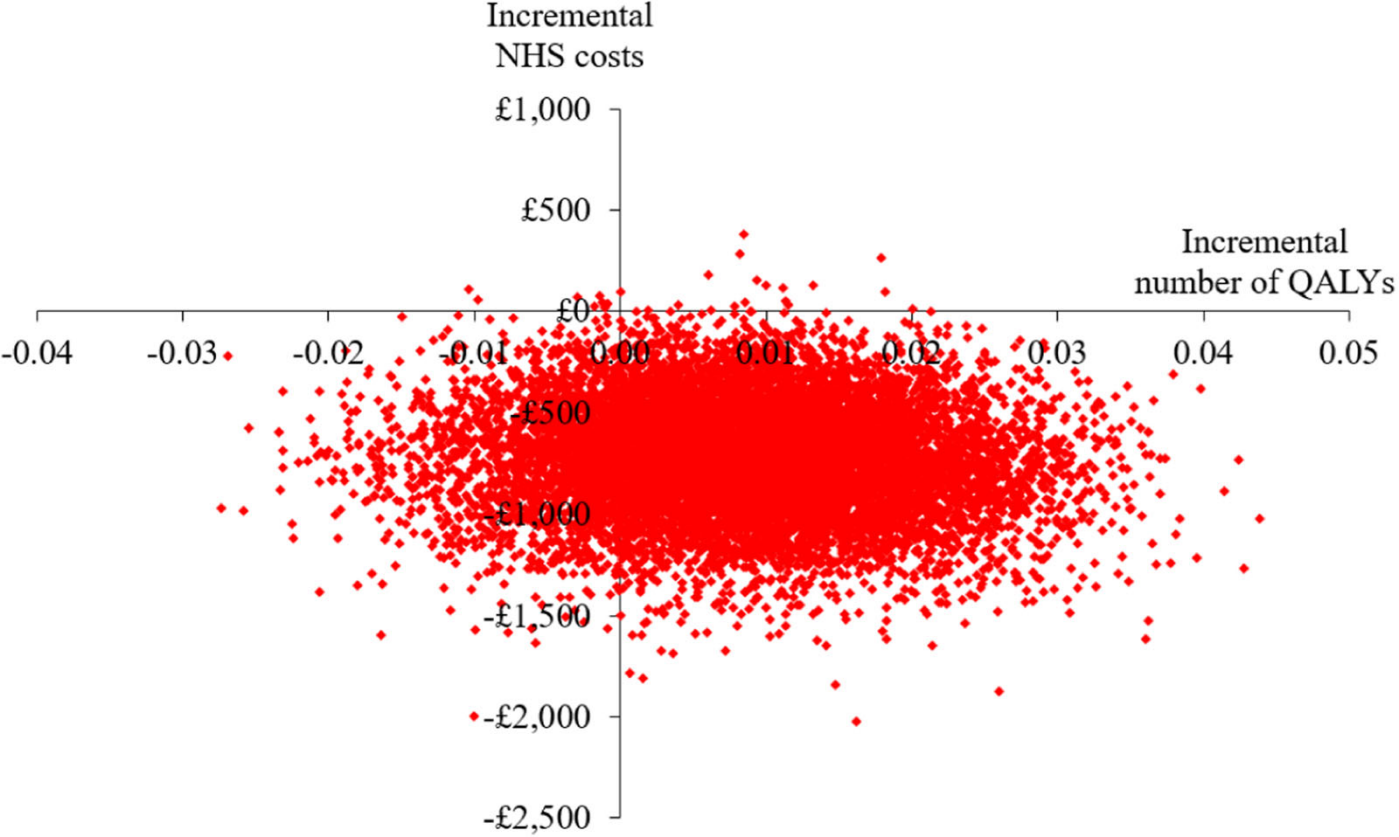
Scatterplot of the incremental cost‐effectiveness of adjunctive chloramine compared with standard care alone following 10,000 iterations of the model.

Figure 3 highlights the distribution in the incremental costs and QALYs between the two groups. The bottom right‐hand (dominant) quadrant in the graph contains proportionally more of the samples. Furthermore, outputs from the PSA indicated that at a cost‐effectiveness threshold of £20,000 per QALY, up to 91% of a cohort would be treated cost‐effectively with adjunctive chloramine, compared with standard care alone.

#### 3.3.2 Deterministic sensitivity analysis

**Figure 4:**
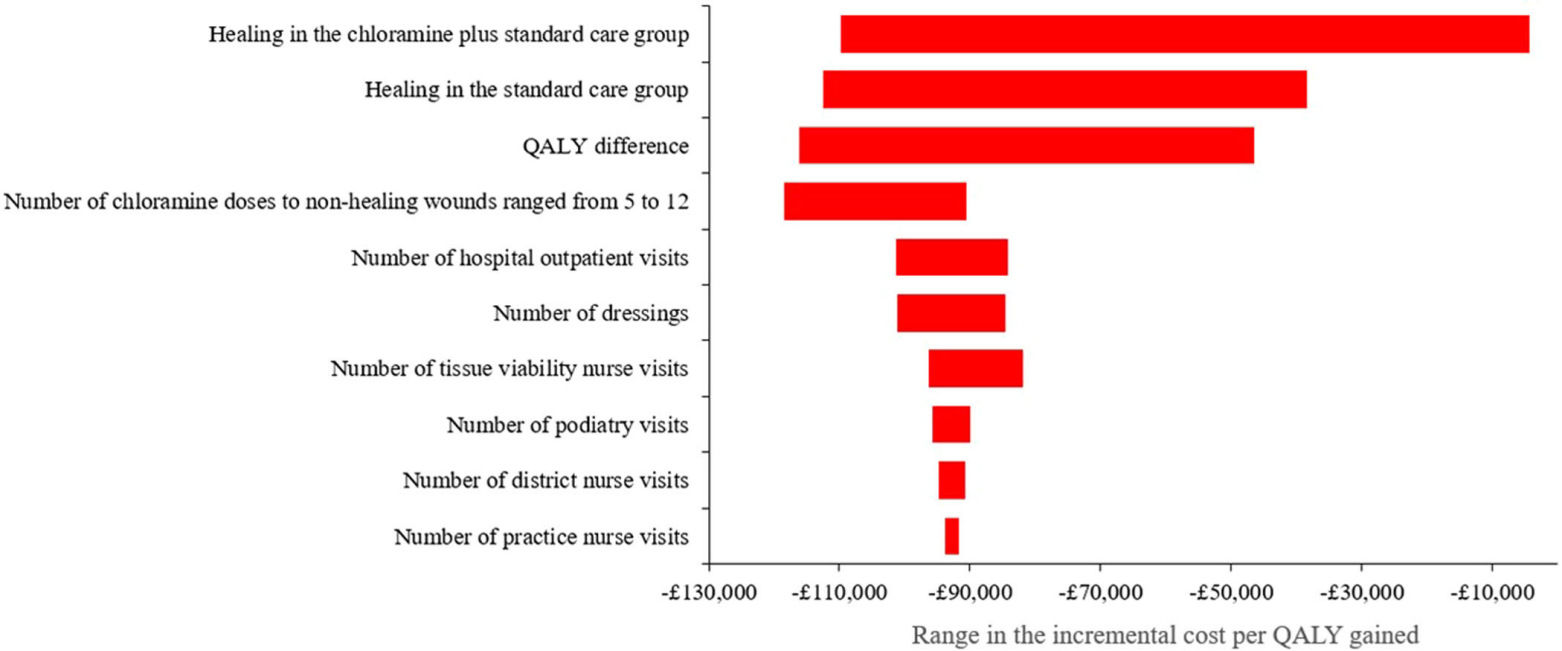
Tornado diagram showing the influence of varying the base case values of key variables by ±20%, unless otherwise stated, on the relative cost‐effectiveness of adjunctive chloramine.

The tornado diagram (Figure 4) showed that adjunctive chloramine's cost‐effectiveness is potentially sensitive to:

- Healing rates in both groups.
- Distribution of clinicians who manage the ulcers.
- QALY difference between the groups.
- Cost of chloramine.

This analysis showed that even if the base case values of these variables change by ±20%, the incremental cost per QALY gained would still be <£20,000. Consequently, adjunctive chloramine would continue to afford the UK's health services a cost‐effective intervention. Changes to the other model inputs had minimal effect on the relative cost‐effectiveness of adjunctive chloramine over the time horizon of the model.

The distribution of healthcare professionals who will administer the chloramine gel in clinical practice is currently unknown. Nevertheless, sensitivity analysis indicated that adjunctive chloramine would remain a dominant treatment irrespective of the distribution of healthcare professionals who manage the ulcers. (Table 6).

**Table 6:** Sensitivity of changing the ratio of healthcare professionals who manage the ulcers.

	Practice nurses	District nurses	Tissue viability nurses	Podiatrists	Hospital outpatient nurses	Incremental cost per QALY gained
Base case	20%	20%	20%	20%	20%	−£92,875
Scenario 1	5%	5%	5%	5%	80%	−£184,125
Scenario 2	25%	25%	25%	25%	0%	−£62,375
Scenario 3	35%	50%	5%	5%	5%	−£56,000
Scenario 4	50%	50%	0%	0%	0%	−£41,125
Scenario 5	100%	0%	0%	0%	0%	−£54,750
Scenario 6	0%	100%	0%	0%	0%	−£27,375
Scenario 7	0%	0%	0%	0%	100%	−£214,500

## 4 DISCUSSION

DFUs are complex wounds that present significant challenges to clinicians, and cost‐effective management and healing of these ulcers remain challenging. Indeed, the UK's health services were estimated to be managing around 330,000 DFUs in 2017/18 at an estimated uprated cost of £2.65 billion at 2021/22 prices.^26^ Effective debridement is critical to healing because it enables removal of devitalised and necrotic tissue and biofilm, and a range of both mechanical and nonmechanical debridement methods are available.^12^ However, a recent systematic review of debridement of DFUs reported that the published evidence for comparative effectiveness between different methods of debridement was of low quality due to impaired study methodology. Hence, the choice of debridement at the present time is generally based on available expertise, patient preferences, the clinical context and cost.^27^ Furthermore, very few economic studies on debridement of the diabetic foot are available in the published literature. One such study found clostridial collagenase ointment to be a cost‐effective adjunctive therapy compared with serial sharp debridement.^28^

Against this background, the clinical evidence indicates that adjunctive chloramine is efficacious in the early phase of treating infected DFUs.^21^ Even though there was a minimal difference in the healing rate at 24 weeks,^21^ there was a 36% reduction in the mean time to healing which, according to this economic analysis, would translate into a 13% reduction in the total cost of ulcer management, since a healed wound no longer incurs the use of any healthcare resources. Consequently, this study indicated that adjunctive chloramine has the potential to afford a cost‐effective debridement strategy for infected, non‐healing DFUs to the UK's health services. Furthermore, sensitivity analyses indicated that adjunctive chloramine would remain a potential cost‐effective debridement strategy for plausible changes in the base case values of the model's key variables. Such findings should help inform healthcare professionals on deciding whether to use the gel within their own clinical practice when it becomes available.

This study's model was based on the ITT cohort of patients who were recruited into the Swedish RCT.^21^ Data from the RCT were used to inform the economic model since it was the only study comparing adjunctive chloramine with standard care alone in the management of DFUs at the time of performing this analysis. The advantage of using this RCT for the modelling was that the efficacy and safety of the two treatments were measured under controlled conditions, since there were no differences in baseline parameters or ulcer characteristics between the two groups. Furthermore, none of the patients' baseline variables affected the decrease in ulcer area during the RCT.^21^ At the end of 24 weeks around half of all the patients in the ITT cohort had a healed ulcer.^21^ However, the RCT was an open design and of limited size and different dressings were used, depending on the clinical status of the ulcer at the time of a dressing change. Consequently, the extent to which the findings from this single RCT would be reproduced in other settings is unknown. Moreover, the patients who participated in the RCT may not be representative of patients with a DFU in the UK.

It is not possible to generalise the results of this economic analysis to patients with other wound types because the modelling was based on the results of a single RCT in DFUs. However, adjunctive chloramine has been shown to be efficacious^20^ and cost‐effective^29^ in treating non‐healing venous leg ulcers. Furthermore, the clinical effectiveness of adjunctive chloramine should be similar in comparable patients in other countries, if the care pathways are consistent between the countries. However, it cannot be assumed that adjunctive chloramine's cost‐effectiveness reported in this study would be the same in other countries if those economies used different care pathways or reimbursement mechanisms to those in the UK.

The analysis is limited to a time horizon of 24 weeks and does not consider the potential impact of managing unhealed ulcers beyond that period. Additionally, the probability of a patient developing a second ulcer at a different location to the DFU being evaluated in this analysis or a healed ulcer recurring in the same location are not represented in the model. Such ulcers would be considered a new wound and would enter the model at the start and receive treatment with either adjunctive chloramine or standard care.

There are several other limitations that need to be considered. The RCT did not record patients’ health preferences, consequently published utilities for DFU health states were used. Limitations of this approach are that the published scores may have been derived from subjects who were potentially different to the modelled population or the characteristics of the published health states may differ to those in the model.^22^ However, irrespective of the base case utility scores that were used in the model, sensitivity analyses showed that adjunctive chloramine remained a cost‐effective debriding agent even when the QALY difference between the two groups was varied by ±20%. The model assumed that the frequency of clinician visits and the standard care that patients received in the Swedish trial would be comparable to that received for ulcer management in clinical practice in the UK. The impact of these limitations and the consequential uncertainty have been assessed, to some extent, by our extensive sensitivity analyses. Nevertheless, the findings from this economic analysis should be informed with the outcomes from a controlled study in the UK.

The analysis was unable to consider the effect of some other factors which may have influenced the results, such as underlying disease severity. Direct costs incurred by patients and indirect societal costs as a result of absenteeism from work have been excluded from the model, which focused exclusively on the perspective of the payers. Consequently, the relative cost‐effectiveness of adjunctive chloramine may have been underestimated. Furthermore, the analysis was unable to consider whether treatment with chloramine conferred any intangible benefits to patients, irrespective of whether their ulcer healed. Additionally, the analysis was unable to evaluate a clinician's skills in applying chloramine or determine the challenges clinicians may have in debriding infected, non‐healing DFUs in the community.

In conclusion, within the study's limitations, treatment with adjunctive chloramine instead of standard care alone could potentially afford the UK's health services a cost‐effective debridement strategy for infected, non‐healing DFUs, due to its ability to accelerate the time to healing.

## AUTHOR CONTRIBUTIONS

Julian F Guest: Conceptualisation; Data curation; Formal analysis; Funding acquisition; Methodology; Validation; Writing—original draft; Writing—review and editing. Björn Eliasson: Data curation; Validation; Writing—review and editing.

## CONFLICT OF INTEREST STATEMENT

The authors declare no conflicts of interest.

## TRANSPARENCY STATEMENT

The lead author Julian F. Guest affirms that this manuscript is an honest, accurate, and transparent account of the study being reported; that no important aspects of the study have been omitted; and that any discrepancies from the study as planned (and, if relevant, registered) have been explained.

## Data Availability

All data relevant to the study are included in the article. Questions concerning the data underlying the results can be sent to the corresponding author.
